# An Analysis of Factors Linked to the Decline in Maternal Mortality in Nepal

**DOI:** 10.1371/journal.pone.0093029

**Published:** 2014-04-04

**Authors:** Sanu Shrestha, Jacqueline S. Bell, Debbi Marais

**Affiliations:** Division of Applied Health Sciences, University of Aberdeen, Scotland, United Kingdom; Aga Khan University, Pakistan

## Abstract

Nepal experienced a steep decline in maternal mortality between 1996 and 2006, which had again dropped by 2010. The aim of this study was to investigate any trends in factors that may be responsible for this decline. The study was based on a secondary data analysis of maternity care services and socio-demographic variables extracted from the Nepal Demographic Health Surveys (1996, 2001, 2006 and 2011). Complex sample analysis was performed to determine the trends in these variables across the four surveys. Univariate logistic regression was performed for selected maternity care service variables to calculate the average change in odds ratio for each survey. Multivariate logistic regression was performed to determine the trends in the health service uptake adjusting for socio-demographic variables. There were major demographic and socio-economic changes observed between 1996 and 2011: notably fewer women delivering at ‘high risk’ ages, decreased fertility, higher education levels and migration to urban areas. Significant trends were observed for improved uptake of all maternity care services. The largest increase was observed in health facility delivery (odds ratio = 2.21; 95% confidence interval = 1.92, 2.34) and women making four or more antenatal visits (odds ratio = 2.24; 95% confidence interval = 2.03, 2.47). After adjusting for all socio-demographic factors, the trends were still significant but disparities become more pronounced at the extremes of the socio-economic spectrum. The odds ratios for each maternity care service examined decreased slightly after adjusting for education, indicating that improved levels of education could partly explain these trends. The improved utilisation of maternity care services seems essential to the decline in maternal mortality in Nepal. These findings have implications for policy planning in terms of government resources for maternity care services and the education sector.

## Introduction

Maternal mortality is a sensitive indicator of disparity that reflects the status of women [1], [2]. Despite the efforts of the international community in controlling maternal mortality, it remains a leading cause of death worldwide among women of reproductive age [3] and a global public health problem [1], [4], especially in developing countries [2], [5]. Globally, an estimated 300,000 maternal deaths occur annually owing to causes associated with pregnancy, of which nearly 75% are preventable [4]. Ninety-nine percent of global maternal deaths occur in developing countries, and of these sub-Saharan Africa and South Asia account for nearly 87% [6].

However the situation is slowly improving, and maternal mortality is declining globally at an annual rate of 3.1%, with the highest decline observed in south-eastern Asia where the maternal mortality ratio (MMR) dropped from 590 to 220 maternal deaths per 100,000 live births between 1990 and 2010 [7]. In order to achieve the fifth Millennium Development Goal (MDG 5) of reducing maternal mortality by three-quarters between 1990 and 2015, an annual decline of 5.5% is needed [8]. Taking the reported rates into consideration, it seems that achievement of MDG 5 is doubtful unless lessons learnt from those countries achieving the most substantial reductions can be shared and implemented.

Nepal is an economically poor country with a human development index of nearly 0.42, which ranks 138 among 169 countries and has a population of nearly 27.5 million [9]. Nearly 88% of the population live in rural areas and 44% of households live below the poverty line [10]. Nepal, like many other developing nations, faces the same challenges of high child and maternal mortality, malnutrition and low life expectancy [11]. Despite these challenges, Nepal has experienced a decline in maternal mortality, with the MMR almost halving between 1996 and 2006 from 539 to 281 per 100,000 live births [12]. The latest estimates suggest that Nepal had already achieved 78% of the MDG-5 target by 2010 (170 per million live births) [7]. It is important to note that the Nepalese MMR is relatively low in comparison to other South-east Asian countries with the exception of the Philippines which have a lower MMR than Nepal [13]. Although the MMR is decreasing, evidence indicates that 67% of maternal mortality in Nepal occurs outside the health facilities [10] and 69% results from direct causes [14].

Hussein and colleagues illustrated the declining trend of maternal mortality in Nepal and identified a number of possible factors that could explain the reduction. The improvements in the coverage of care (delivery care by health professionals in a health facility, met need for emergency obstetric care and Caesarean section) were hypothesized as potential drivers [12].

Delivery with a skilled birth attendant (SBA), along with the provision of essential equipment, drugs and supplies in order to cope with obstetric complications, is regarded as one of the important strategies to prevent maternal deaths [15], [16]. Skilled health personnel can provide interventions to prevent and manage life-threatening complications when optimum care is required [17]. The proportion of deliveries with skilled attendants increased globally from 55% to 65% between 1990 and 2009 [7]. Dramatic progress was observed in southern Asia as the proportion of births attended by skilled birth attendants rose from 32% to 50% over the same period [17].

Health facility delivery is also essential for reducing maternal deaths resulting from complications of late pregnancy and labour [18]. Women delivering in a clean and safe environment along with the presence of a SBA have a reduced risk of maternal and newborn morbidity and mortality [19]. The proportion of health facility deliveries and Caesarean section (CS) deliveries are indicators of emergency obstetric and newborn care (EmONC), and are important in preventing maternal mortality [20], [21]. There has been a considerable increase in CS, particularly among urban residents of developing countries [22].

Universal access to antenatal care (ANC) is a priority too [23]. ANC provides an opportunity for the women to seek skilled care during delivery and to encourage women to seek healthcare services to improve both maternal and neonatal health [24]. Regular antenatal visits are beneficial for identifying and preventing unfavourable pregnancy outcomes, provided that they are initiated at an early stage of pregnancy and continued throughout until delivery [18], [25]. The percentage of women having at least one antenatal visit with a skilled professional has increased globally from 64% to 81% from 1990 to 2010 [7]. In developing countries, the proportion of women having four or more antenatal visits increased substantially from 35% to 51% between 1990 and 2009 [17]. Evidence suggests that the timing of the first antenatal visit is an important factor influencing delivery care. Women initiating an early ANC visit had a higher probability of using skilled attendance during delivery compared with their peers who had their first visit at a later stage of pregnancy [26].

The objectives of the study were to: investigate the trends in factors that may be linked to the decline in maternal mortality in Nepal and determine the trends in the health services uptake adjusting for socio-demographic variables.

## Methodology

The study is based on secondary data analysis of maternity care services and socio-demographic variables extracted from the Nepal Demographic and Health Surveys (NDHS) from 1996, 2001, 2006 and 2011. The NDHS are nationally representative household surveys of women of reproductive age (15–49 years). The purpose of the surveys is to provide detailed information to policy makers on the levels and determinants of fertility; family planning; infant, child, adult and maternal mortality; maternal and child health; women’s empowerment; nutrition; and knowledge of HIV/AIDS.

To reduce information and selection biases in the surveys, the sampling design included representative proportions from rural and urban regions, the three ecological zones and five development regions in Nepal. The sample was selected on the basis of two-stage stratified sampling of the households. Initially, primary sampling units (PSU) were selected by a systematic sampling technique. Household listings were then carried out for each selected PSU to provide a sampling frame for the second stage of sampling. The NDHS interviewed women reporting at least one live birth in the preceding five years. For women who reported more than one delivery, only their most recent delivery was included in the analysis.

### Maternity Care Services Variables

Variables included were: place of delivery (whether it occurred at a health facility or outside the health facility), attendance by a health professional (as a proxy for SBA) at delivery, type of delivery (CS or vaginal) and several ANC practices (whether ANC was provided by a health professional; whether the women had made four or more visits and whether the first antenatal visit was within first trimester of pregnancy).

### Socio-demographic Variables

Socio-demographic variables included in the analysis were: mother’s age at delivery (<20 years, 20–30 years, 31 years or above), mother’s highest education level (no education, primary, secondary and above); parity (1, 2–4); area of residence (rural, urban); Employment status (no, yes); Region (Central, Eastern, Western, Mid-Western, Far-Western); Ecological Zones (mountain, hill, terai); Religion (Hindu, Buddhist, Muslim, Christian, Others) and Ethnicity (Brahmin, Chhetri, Newars, Janajati, Dalit, Others). The wealth indices in the NDHS surveys were computed using principal components analysis to generate an asset score based on information available in the household questionnaire, including the source of energy within the household, toilet facilities and the availability of television, telephone and electricity. The score produced was divided into quintile groups from poorest to wealthiest. In this way women were categorized on the basis of total wealth within the household [27].

### Statistical Analysis

Statistical analysis was performed using SPSS v20 and all four datasets were combined into a single dataset to facilitate further analysis. Complex sample analysis was used to take into account the clustering of data and sample weights were used to adjust for differences in the probability of selection to ensure that results were representative at a national level.

Socio-demographic factors were tabulated for each survey and trends in percentages for each variable category across the four surveys were identified using year of survey as the independent variable in a linear regression. Similarly, the percentages of women using maternity services were presented graphically and univariate logistic regression was conducted for each maternity care variable using year of survey as the independent variable. Lastly, multivariate logistic regression was performed to determine the trends in the maternity services uptake adjusting for socio-demographic variables.

## Results

A total of 18,130 women from four NDHS were included in the analysis - 1996 (N = 4402); 2001 (N = 5514); 2006 (N = 4066); 2011 (N = 4148). There were major demographic and socio-economic changes observed over the period (Table 1). There was a significant increase from 72.5% to 83.5% in the proportion of women delivering between the ages of 20–30 years, with fewer women delivering at high risk ages (<20 and ≥35 years). Fertility dropped gradually significantly as the proportion of women delivering higher order births decreased from 72.9% to 68.6%. The proportion of women having attended at least secondary school increased nearly four-fold from 9.7% to 36.0%. Similarly, a movement to residence in urban areas and employment for women were both significant trends. Additionally, changes in the distributions of religion, ethnicity, ecological zones, region and wealth quintile were all found to be statistically significant (Table 1).

**Table 1:** Socio-demographic trends between 1996 and 2011.

Characteristic	1996	2001	2006	2011	P-value for trend
	(N = 4402)	(N = 5514)	(N = 4066)	(N = 4148)	
**Age at delivery**					
Less than 20	13.1%	13.0%	8.8%	8.7%	<0.001
20–34	72.5%	74.0%	80.3%	83.5%	<0.001
35 and above	14.4%	13.0%	10.9%	7.8%	0.006
**Parity**					
1^st^	27.0%	26.7%	26.9%	31.4%	<0.001
2 to 4	72.9%	73.3%	73.1%	68.6%	<0.001
**Highest education level**					
No education	77.7%	70.7%	58.0%	43.9%	<0.001
Primary	12.6%	15.5%	18.3%	20.1%	<0.001
Secondary and above	9.7%	13.8%	23.8%	36.0%	<0.001
**Type of place of residence**					
Urban	6.5%	7.0%	13.2%	10.1%	<0.001
Rural	93.5%	93.0%	86.8%	89.9%	<0.001
**Quintile of wealth index**					
Lowest	24.9%	24.2%	23.5%	23.6%	0.107
Second	20.4%	20.7%	21.1%	21.7%	0.128
Middle	21.2%	20.0%	20.0%	21.0%	0.878
Fourth	19.2%	20.2%	18.5%	18.0%	0.051
Highest	14.3%	14.9%	16.9%	15.7%	0.013
**Employment**					
Yes	74.4%	81.0%	69.6%	16.1%	<0.001
No	25.6%	19.0%	30.4%	43.9%	<0.001
**Region**					
Eastern	21.0%	23.6%	21.7%	24.1%	0.013
Central	33.4%	32.5%	32.7%	31.2%	0.046
Western	19.8%	19.1%	18.6%	23.4%	<0.001
Mid western	15.8%	14.5%	12.6%	12.4%	<0.001
Far western	10.0%	10.3%	14.4%	8.9%	0.648
**Ecological zones**					
Mountain	7.4%	7.3%	8.4%	7.4%	0.615
Hill	42.0%	40.6%	41.2%	40.2%	0.189
Terai	50.6%	52.1%	50.4%	52.4%	0.304
**Religion**					
Hindu	86.0%	84.6%	84.9%	83.0%	0.001
Buddhist	6.5%	7.1%	7.7%	8.7%	<0.001
Muslim	6.0%	5.6%	4.8%	5.7%	0.229
Christian	0.3%	0.8%	0.8%	1.2%	<0.001
Others	1.2%	1.9%	1.8%	1.4%	0.706
**Ethnicity**					
Brahmin	11.1%	9.4%	10.1%	11.8%	0.191
Chhetri	17.2%	15.1%	17.6%	19.1%	0.001
Newar	4.6%	3.9%	3.5%	3.1%	<0.001
Janajati	28.5%	30.6%	33.2%	33.6%	<0.001
Dalit	15.6%	19.1%	18.5%	16.5%	0.469
Other	23.0%	21.9%	17.2%	15.9%	<0.001

**Figure 1:**
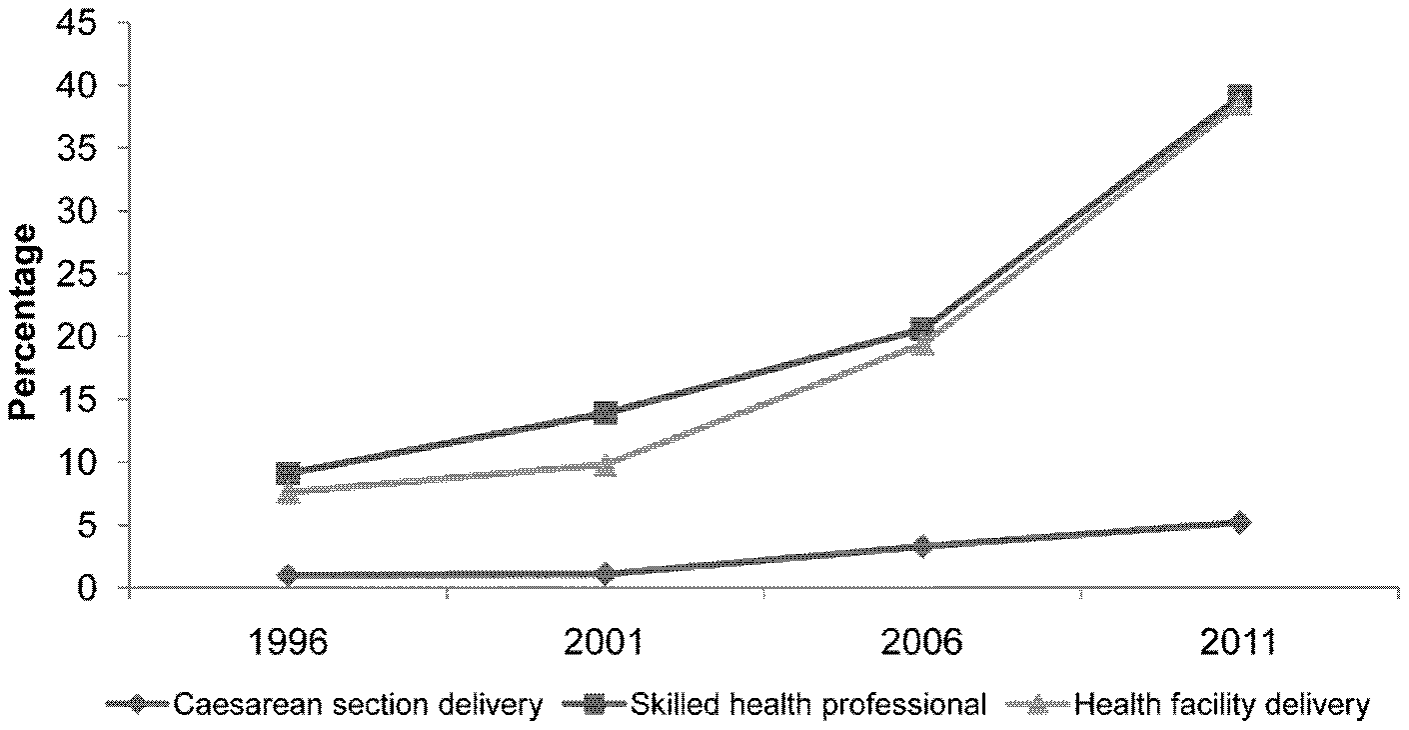
Trends in delivery care between 1996 and 2011. The percentage of the women delivering in health facilities increased slightly between 1996 and 2001, and nearly doubled in 2006. In 2006, 19.4% of respondents delivered in a health facility, which increased dramatically to 38.5% in 2011. The assistance during delivery by skilled birth attendants increased marginally from 9.1% to 20.6% between 1996 and 2006. However, it rose significantly to 39.1% in 2011. Women reporting Caesarean section delivery increased slightly from 1% in 1996 to 1.1% in 2001. It then increased drastically to 5.2% in 2011.

**Figure 2:**
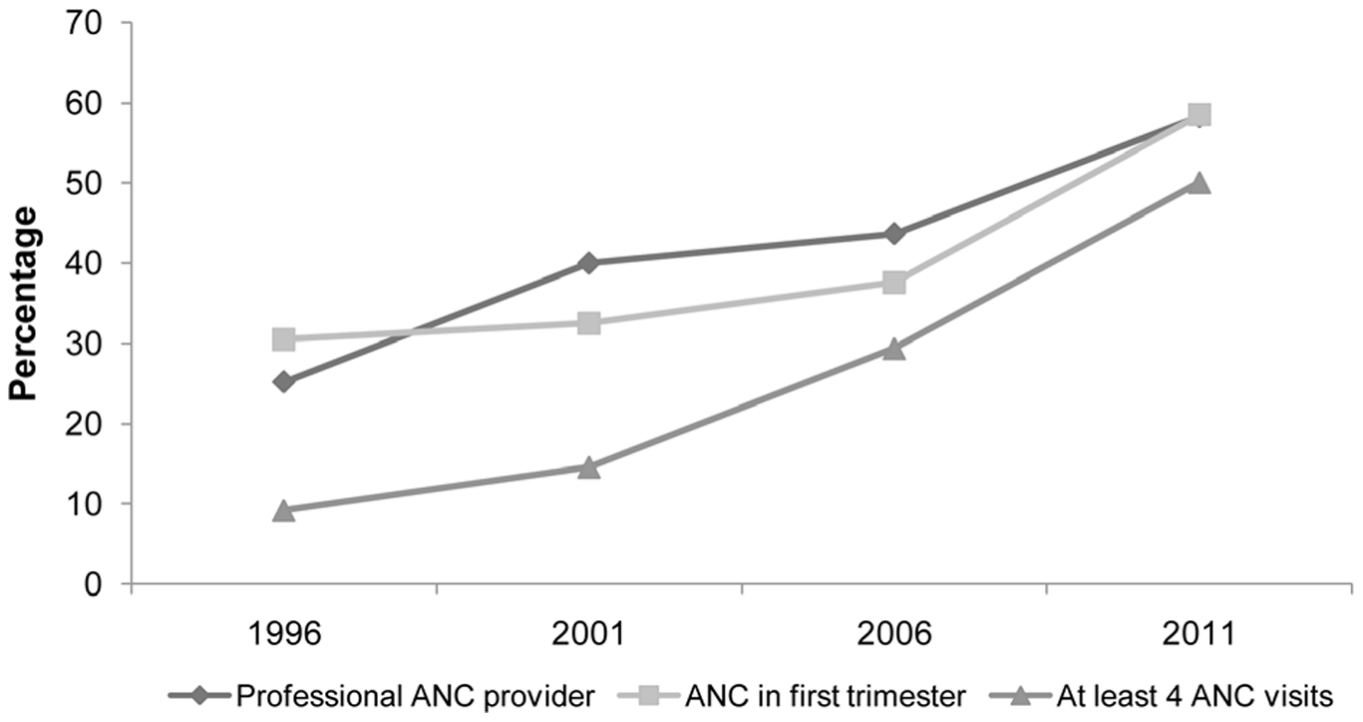
Trends in antenatal care between 1996 and 2011. Antenatal care (ANC) from skilled professionals nearly doubled over time. In 1996 only 25.2% of the pregnant women received ANC from a skilled professional, but increased to 58.3% in 2011. The proportion of women with four or more ANC visits increased significantly from 9.2% to 50.1% between 1996 and 2011. The results from the surveys indicated that an antenatal check-up within the first trimester nearly doubled from 30.5% in 1996 to 58.5% in 2011.

All the maternity services examined showed large statistically significant increases in uptake over the study period. The percentage of the women delivering in health facilities increased slightly between 1996 (7.6%) and 2001 (9.8%), but had nearly doubled by 2006 (19.4%) and saw a similar rise to 38.5% in 2011 (Figure 1). Assistance during delivery by a health professional mirrored these changes, doubling from 9.1% in 1996 to 20.6% in 2006 and rising to 39.1% in 2011. Over the whole period women reporting CS delivery increased to 5.2% in 2011 from 1.0% in 1996 (Figure 1). In antenatal care between 1996 and 2011: check-ups within the first trimester nearly doubled from 30.5% to 58.5%; the percentage of women attending four or more visits increased from 9.2% to 50.1%; and ANC from professionals nearly doubled from 25.2% to 58.3% (Figure 2).

Univariate logistic regression indicates that these improvements in maternity care practices were all statistically significant (p<0.001) (Table 2). The greatest increase was observed in health facility delivery (OR = 2.21; 95% CI = 1.92, 2.34) and women making four or more antenatal visits (OR = 2.24; 95% CI = 2.03, 2.47).

**Table 2:** Significant trends between 1996 and 2011 in maternity care services.

Variable	Crude odds ratio (OR)	Confidence- interval (CI)	P value
**Assistance during delivery:** Skilled birth attendance	1.892	1.731–2.067	<0.001
**Place of delivery:** Health facility	2.213	1.925–2.341	<0.001
**Delivery:** Caesarean section	1.911	1.662–2.198	<0.001
**ANC provider:** Skilled health professional	1.541	1.421–1.671	<0.001
**Number of ANC visits:** Four or more visits	2.247	2.038–2.478	<0.001
**Timing of ANC check-up:** First trimester of pregnancy	1.516	1.425–1.612	<0.001

*Note*: Crude odds ratio is the average change per each survey, assuming the trend across the survey is linear.

ANC - antenatal care.

Multivariate analysis after adjusting for socio-demographic factors shows a significant average increase in health facility delivery, delivery assisted by a professional and CS delivery across all the surveys (Table 3). Disparities become more pronounced at the extremes of the socio-economic spectrum. Health facility delivery, professional assistance during delivery and CS delivery were more common among urban women, women with at least secondary school education, and the highest wealth quintile respectively. However, higher parity and employed women were less likely to deliver in a health facility or assisted by a professional. Although highly educated women were more likely to deliver in a health facility and in the presence of a professional, they were less likely to have CS delivery. Similarly, increases in ANC with a health professional, four or more ANC visits and antenatal check-up within the first trimester of pregnancy continued to show significant improvements after adjusting for socio-demographic factors (Table 4). Urban/rural, poor/rich and uneducated/highly educated disparities in ANC care were substantial, with higher parity, older and employed women less likely to receive ANC.

**Table 3:** Association between socio-demographic factors and delivery variables.

	Delivery in a healthfacility OR (95% CI)	Professional assistanceat delivery OR (95% CI)	Caesarean sectionOR (95% CI)
**Year**			
1996	1.00	1.00	1.00
2001	1.34 (1.06–1.69)**	1.73 (1.38–2.17)*	1.02 (0.63–1.63)
2006	2.68 (2.12–3.40)*	2.26 (1.78–2.88)*	2.72 (1.69–4.39)*
2011	7.69 (5.82–10.16)*	6.31 (4.80–8.29)*	3.74 (2.28–6.13)*
**Survey trend**	2.01 (1.83–2.21)*	1.76 (1.61–1.92)*	1.68 (1.41–2.00)*
**Age at delivery (yrs)**			
<20	1.00	1.00	1.00
20–34	1.51 (1.20–1.90)*	1.35 (1.09–1.68)**	1.82 (1.19–2.77)**
≥35 or more	2.09 (1.47–2.96)*	1.78 (1.28–2.46)**	3.80 (1.90–7.61)*
**Birth order**			
1^st^	1.00	1.00	1.00
2–4	0.32 (0.27–0.38)*	0.33 (0.28–0.39)*	0.27 (0.19–0.40)*
**Education**			
No education	1.00	1.00	1.00
Primary education	1.54 (1.20–1.97)**	1.68 (1.36–2.09)*	1.16 (0.75–1.78)
Secondary and above	2.88 (2.32–3.58)*	3.29 (2.72–3.98)*	0.90 (0.55–1.47)
**Wealth Quintile**			
Lowest	1.00	1.00	1.00
Second	1.75 (1.28–2.41)*	1.66 (1.26–2.18)*	0.79 (0.31–2.00)
Middle	2.13 (1.53–2.99)*	2.02 (1.48–2.75)*	1.57 (0.66–3.71)
Fourth	2.94 (2.12–4.08)*	2.83 (2.10–3.82)*	2.66 (1.19–5.90)**
Highest	6.9 (4.76–10.03)*	6.69 (4.82–9.29)*	5.27 (2.23–12.46)*
**Respondent working**			
No	1.00	1.00	1.00
Yes	0.77 (0.64–0.91)**	0.73 (0.62–0.87)*	0.71 (0.52–0.99)**
**Residence**			
Rural	1.00	1.00	1.00
Urban	2.62 (2.09–3.28)*	2.53 (2.08–3.09)*	1.69 (1.21–2.36)**
**Ecological zones**			
Mountain	1.00	1.00	1.00
Hill	1.83 (1.32–2.56)*	1.89 (1.41–2.53)*	2.40 (1.09–5.26)**
Terai	1.73 (1.24–2.43)**	1.83 (1.36–2.46)*	2.95 (1.31–6.62)**
**Region**			
Far-western	1.00	1.00	1.00
Mid-western	1.02 (0.69–1.52)	0.97 (0.68–1.39)	1.23 (0.67–2.27)
Western	0.92 (0.63–1.33)	0.87 (0.62–1.23)	1.56 (0.83–2.91)
Central	1.37 (0.95–1.98)	1.23 (0.89–1.70)	1.59 (0.87–2.89)
Eastern	1.13 (0.78–1.61)	1.27 (0.91–1.75)	1.55 (0.88–2.71)
**Religion**			
Hindu	1.00	1.00	1.00
Buddhist	1.16 (0.78–1.73)	1.16 (0.79–1.70)	2.08 (1.04–4.18)**
Muslim	0.93 (0.59–1.45)	0.89 (0.60–1.31)	0.52 (0.17–1.64)
Christian	0.43 (0.19–0.98)**	0.64 (0.33–1.24)	0.56 (0.11–2.77)
Others	0.83 (0.43–1.60)	0.80 (0.46–1.40)	0.64 (0.13–3.02)
**Ethnicity**			
Janajati	1.00	1.00	1.00
Others	1.34 (0.96–1.85)	1.41 (1.02–1.93)**	1.90 (1.11–3.25)**
Dalit	1.27 (0.94–1.72)	1.24 (0.93–1.65)	1.43 (0.79–2.58)
Newar	2.68 (2.01–3.57)	2.47 (1.84–3.33)*	1.98 (1.17–3.33)**
Chhetri	1.61 (1.23–2.10)**	1.56 (1.21–2.01)**	2.00 (1.18–3.38)**
Brahmin	1.88 (1.42–2.48)*	1.85 (1.43–2.39)*	2.27 (1.37–3.75)**

*P<0.001,

**P<0.05, OR: odds ratio, CI: confidence interval.

**Table 4:** Association between socio-demographic factors and ANC variables.

	ANC provided by healthprofessional OR (95% CI)	Four or more ANCvisits OR (95% CI)	First ANC visit in firsttrimester OR (95% CI)
**Year**			
1996	1.00	1.00	1.00
2001	2.38 (1.97–2.86)*	1.84 (1.46–2.31)*	1.07 (0.89–1.29)
2006	2.22 (1.74–2.83)*	4.08 (3.06–5.45)*	1.30 (1.08–1.58)**
2011	3.68 (2.82–4.80)*	9.8 (7.21–13.31)*	2.96 (2.42–3.63)*
**Survey trend**	1.45 (1.33–1.58)*	2.16 (1.95–2.39)*	1.40 (1.31–1.50)*
**Age at delivery (yrs)**			
<20	1.00	1.00	1.00
20–34	1.04 (0.88–1.24)	1.34 (1.12–1.59)**	1.43 (1.18–1.74)*
≥35 or more	0.61 (0.48–0.77)*	0.69 (0.50–0.95)**	1.68 (1.26–2.23)*
**Birth order**			
1^st^	1.00	1.00	1.00
2–4	0.62 (0.54–0.71)*	0.51 (0.43–0.60)*	0.75 (0.65–0.87)*
**Education**			
No education	1.00	1.00	1.00
Primary education	1.80 (1.53–2.12)*	2.03 (1.71–2.40)*	1.11 (0.93–1.31)
Secondary and above	2.83 (2.38–3.35)*	3.27 (2.72–3.92)*	1.64 (1.40–1.92)*
**Wealth Quintile**			
Lowest	1.00	1.00	1.00
Second	1.47 (1.25–1.72)*	1.45 (1.18–1.79)*	1.01 (0.84–1.23)
Middle	1.89 (1.58–2.25)*	2.06 (1.64–2.57)*	1.08 (0.87–1.32)
Fourth	2.64 (2.17–3.21)*	2.58 (2.03–3.29)*	1.11 (0.90–1.380
Highest	5.47 (4.21–7.10)*	4.29 (3.22–5.71)*	1.55 (1.22–1.96)*
**Respondent working**			
No	1.00	1.00	1.00
Yes	0.75 (0.64–0.88)**	0.84 (0.72–0.97)**	0.95 (0.83–1.08)
**Residence**			
Rural	1.00	1.00	1.00
Urban	2.93 (2.29–3.75)*	1.56 (1.23–1.97)*	1.45 (1.21–1.74)*
**Ecological zones**			
Mountain	1.00	1.00	1.00
Hill	0.62 (0.54–0.71)	1.25 (0.90–1.72)	1.10 (0.90–1.35)
Terai	1.12 (0.82–1.53)	1.36 (0.99–1.88)	0.94 (0.75–1.17)
**Region**			
Far-western	1.00	1.00	1.00
Mid-western	0.97 (0.64–1.46)	0.61 (0.41–0.92)**	1.43 (1.13–1.80)**
Western	1.47 (0.99–2.17)	0.72 (0.48–1.07)	1.28 (1.01–1.62)**
Central	0.97 (0.65–1.44)	0.69 (0.47–1.01)	1.27 (1.01–1.59)**
Eastern	1.25 (0.85–1.84)	0.73 (0.50–1.08)	1.38 (1.10–1.73)**
**Religion**			
Hindu	1.00	1.00	1.00
Buddhist	1.17 (0.84–1.62)	1.08 (0.78–1.50)	0.96 (0.74–1.26)
Muslim	1.12 (0.71–1.76)	0.91 (0.65–1.28)	0.86 (0.54–1.36)
Christian	1.14 (0.57–2.30)	0.86 (0.39–1.88)	1.03 (0.52–2.03)
Others	1.42 (0.76–2.63)	0.93 (0.59–1.46)	0.61 (0.44–0.84)**
**Ethnicity**			
Janajati	1.00	1.00	1.00
Others	1.35 (0.99–1.84)	0.84 (0.62–1.15)	0.90 (0.71–1.14)
Dalit	1.50 (1.18–1.90)**	1.04 (0.79–1.37)	0.76 (0.62–0.94)**
Newar	2.54 (1.67–3.84)*	3.31 (2.26–4.85)*	1.24 (0.95–1.61)
Chhetri	2.00 (1.55–2.58)*	1.56 (1.16–2.09)**	0.84 (0.69–1.03)
Brahmin	2.81 (2.12–3.72)*	2.00 (1.55–2.58)*	1.10 (0.88–1.36)

*P<0.001,

**P<0.05, OR: odds ratio, CI: confidence interval.

## Discussion

This study has revealed important upward trends in the use of delivery and antenatal services between 1996 and 2011 in Nepal, corroborating the findings in earlier studies [12] and, importantly, indicating that the improvement is continuing. As previously hypothesised, this may account for the decrease in MMR in Nepal to 170 per 100,000 live births in 2010 [7], [12]. Findings also revealed major socio-economic changes especially in terms of the education of women, their employment status and movement to urban areas, as well as positive maternal health behaviours such as fewer and less high risk pregnancies. Lastly, the analysis shows the marked and continuing sub-national variation in uptake of care that are usually summarised at a national or regional level. With this inequity in service uptake, many of the socio-economic changes could be expected to impact on maternal mortality through increased access to health services; however further analysis indicates that socio-economic improvements explain only a small part of the increase in uptake of care: only adjustment for education reduced the upward trend in odds ratios for maternity care. This means that most of the influence of improved socio-economic indicators on maternal health is mediated through other pathways, for example, improved nutrition and general health.

Women with at least secondary education are over three times more likely to deliver with a health professional than women with no education, and the percentage in this category has increased from under 10% in 1996 to 36% in 2011. Increases in the number of educational institutions and teachers and the free education services provided in government-run educational institutions contribute to this rising trend in the educational status of women [28]; and this improvement partly explains the trends in delivery with a professional and delivery in a health facility. Interestingly, employed women have a lower probability of utilizing maternity care services, which is in agreement with a previous study in Nepal [29]. A possible explanation for this is that working women remain busy with their work and hence may not have enough time to seek maternal health care services.

It seems clear that evolving governmental policy and strategies have had a major impact on the improvements in the availability of maternal healthcare services. For example, the Human Resource Strategy for safe delivery and the development of a national policy to assure the presence of SBAs at deliveries wherever they take place have contributed in the rise in the number of deliveries with a professional attendant in Nepal [30]. Indeed, governmental support has resulted in Nepal reaching the MDG target for 60% of births attended by skilled health professionals by 2015. The support of the Ministry of Health and Population in providing financial incentives for health facilities for providing CS delivery could have contributed to the increase in the number of CS deliveries [31], which were shown to have grown five-fold in the past 15 years. Another reason for this significant trend could also be the increase in the number of health facilities providing EmONC and referrals. It has been suggested that a CS rate of 5% is optimum for preventing maternal and neonatal mortality [32] if these deliveries are conducted in response to obstetric complications such as eclampsia, pre-eclampsia and obstructed labour [33]. The government of Nepal had also set a specific MDG target to increase the proportion of women making four or more ANC visits to 80% by 2015 [30], although this seems to be one of the targets that will not be achieved by 2015. These findings are similar to those in Bangladesh where there is also an increasing uptake of antenatal visits; although in Nepal first trimester visits are also increasing, which is in contrast to findings in Bangladesh [34].

There have also been notable successes in initiatives aiming to strengthen the demand for maternity care. To help overcome demand-side barriers, a Maternity Incentives Scheme was introduced nationwide in 2005, providing cash for transportation costs to women giving birth in public health facilities and providing free services for women in districts with low Human Development Index. A national free delivery policy was subsequently launched in 2009 [35]. Other initiatives have successfully used mass media to disseminate safe motherhood messages and community mobilization to improve uptake of services [36], [37].

Government support is evidently important to reducing maternal mortality and this is one of the lessons for other nations, especially other low and middle income countries. The way forward though is to ensure that the policies and strategies are best suited to the context, cost-effective and sustainable. The findings of this study have raised one such issue which could have major policy implications. The increase in delivery at health facilities was most apparent among the urban, wealthy and highly educated women and CS was more common among urban, wealthy and older women. This is interesting as one of the reasons for this increased uptake is thought to be the implementation of a governmental safe motherhood initiative which provides financial support to women delivering in health facilities [18]. The findings of this study suggest that other possible explanations, including urbanisation and increasing female employment are not the driving force, although this assertion would need to be investigated further. Besides the effort of government, in the context of Nepal, improved family planning and the improved status of women have reduced the death rate of pregnant women [38].

There were several limitations to this study. Ideally we would have modelled how maternal outcome varies with women’s characteristics and service use at an individual level, but we did not have access to suitable data. The NDHS use the sisterhood method to measure maternal mortality (respondents’ report on their siblings’ deaths) so it was not possible to look for a direct association between characteristics/service use and maternal mortality [39]. The choice of socio-demographic factors was based on the literature but was limited by the data collected in the surveys. The logistic regression used describes the average change in effect across all the surveys, which assumes a linear increase in maternity care services. From the trends shown in Figures 1 and 2 it is clear that this assumption did not always hold true, which limits the potential explanatory power of the socio-demographic factors and might partly explain their general lack of impact on observed trends in maternity service uptake.

## Conclusion

The central finding of this study is that the observed improvement in the uptake of maternity care services was largely independent of the improving socio-demographic situation. Of all the factors tested only improvements in women’s educational attainment was shown to have an influence on the improvements in uptake of care. In the context of Nepal, these trends have coincided with a period of steady decline in maternal mortality ratios. The findings have important policy implications indicating that independent improvement in the uptake of maternity care services can have an impact on maternal mortality which could be relevant for other countries having high rates of maternal mortality. The role of governments in upgrading maternity services, and addressing demand-side barriers is essential, and improving the educational status of women is also shown to be an important priority.

## References

[1] Alvarez JL, Gil R, Hernandez V, Gil A (2009) Factors associated with maternal mortality in Sub-Saharan Africa: an ecological study. BMC Public Health 9: 462. 20003411 10.1186/1471-2458-9-462 PMC2801510

[2] Shen C, Williamson JB (1999) Maternal mortality, women’s status, and economic dependency in less developed countries: a cross-national analysis. Soc Sci Med 49 (2): 197–214. 10.1016/s0277-9536(99)00112-4 10414829

[3] Asamoah BO, Moussa KM, Stafstrom M, Musinguzi G (2011) Distribution of causes of maternal mortality among different socio-demographic groups in Ghana; a descriptive study. BMC Public Health 11: 159. 21392387 10.1186/1471-2458-11-159 PMC3063206

[4] Karlsen S, Say L, Souza JP, Hogue CJ, Calles DL, et al (2011) The relationship between maternal education and mortality among women giving birth in health care institutions: analysis of the cross sectional WHO Global Survey on Maternal and Perinatal Health. BMC Public Health 11: 606. 21801399 10.1186/1471-2458-11-606 PMC3162526

[5] Hill K, Thomas K, AbouZahr C, Walker N, Say L, et al (2007) Estimates of maternal mortality worldwide between 1990 and 2005: an assessment of available data. The Lancet 370 (9595): 1311–1319. 10.1016/S0140-6736(07)61572-4 17933645

[6] WHO UNICEF, UNFPA, The World Bank. (2010) Trends in Maternal Mortality: 1990 to 2008.

[7] WHO UNICEF, UNFPA, The World Bank. (2012) Trends in Maternal Mortality: 1990 to 2010.

[8] Shah IH, Say L (2007) Maternal mortality and maternity care from 1990 to 2005: uneven but important gains. Reprod Health Matters 15(30): 17–27. 17938067 10.1016/S0968-8080(07)30339-X

[9] National Planning Commission, United Nations Development Programme (2011) Millennium Development Goals: Needs Assessment for Nepal 2010.

[10] Bhattarai B (2008) Safe motherhood in the context of Nepal. Marriage & Family Review 44(2–3): 318–327.

[11] Pradhan A (2005) Situation of antenatal care and delivery practices. Kathmandu University Medical Journal 3(3): 266–270. 18650590

[12] Hussein J, Bell J, Dar Iang M, Mesko N, Amery J, et al (2011) An appraisal of the maternal mortality decline in Nepal. PLoS ONE 6(5): e19898. 21637836 10.1371/journal.pone.0019898 PMC3102673

[13] Pradhan A, Pant PD, Govindasamy P (2007) Trends in Demographic and Reproductive Health Indicators in Nepal: Further Analysis of the 1996, 2001, and 2006 Demographic and Health Surveys Data. New ERA.

[14] Suvedi BK, Pradhan A, Barnett S, Puri M, Chitrakar SR, et al. (2009) Nepal maternal mortality and morbidity study 2008/2009: summary of preliminary findings. Kathmandu, Nepal: Family Health division, Department of Health Services, Ministry of Health, Government of Nepal.

[15] Bell J, Hussein J, Jentsch B, Scotland G, Bullough C (2003) Improving skilled attendance at delivery: a preliminary report of the SAFE strategy development tool. Birth 30(4): 227–234. 14992153 10.1046/j.1523-536x.2003.00252.x

[16] Van Eijk AM, Bles HM, Odhiambo F, Ayisi JG, Blokland IE, et al (2006) Use of antenatal services and delivery care among women in rural western Kenya: a community based survey. Reproductive Health 3(1): 2. 16597344 10.1186/1742-4755-3-2 PMC1459114

[17] United Nations (2010) The Millennium Development Goals Report 2010.

[18] Ministry of Health and Population [Nepal], New ERA, ICF International Inc. (2012) Nepal Demographic and Health Survey 2011.

[19] Ronsmans C, Graham WJ (2006) Lancet Maternal Survival Series steering group. Maternal mortality: who, when, where, and why. The Lancet 30;368 (9542): 1189–1200. 10.1016/S0140-6736(06)69380-X 17011946

[20] Fauveau V, Donnay F (2006) Can the process indicators for emergency obstetric care assess the progress of maternal mortality reduction programs? An examination of UNFPA Projects 2000–2004. International Journal of Gynecology & Obstetrics 93(3): 308–316. 16682038 10.1016/j.ijgo.2006.01.031

[21] Althabe F, Sosa C, Belizan JM, Gibbons L, Jacquerioz F, et al (2006) Cesarean section rates and maternal and neonatal mortality in low-, medium-, and high-income countries: an ecological study. Birth 33(4): 270–277. 17150064 10.1111/j.1523-536X.2006.00118.x

[22] Stanton CK, Holtz SA (2006) Levels and trends in cesarean birth in the developing world. Stud Fam Plann 37(1): 41–48. 16570729 10.1111/j.1728-4465.2006.00082.x

[23] Urassa DP, Carlstedt A, Nystrom L, Massawe SN, Lindmark G (2002) Quality assessment of the antenatal program for anaemia in rural Tanzania. International Journal for Quality in Health Care 14(6): 441–448. 12515330 10.1093/intqhc/14.6.441

[24] USAID (2011) Focused antenatal care: Providing integrated, individualized care during pregnancy. Available: http://www.accesstohealth.org/toolres/pdfs/accesstechbrief_fanc.pdf. Accessed 2014 Mar 09.

[25] Nuraini E, Parker E (2005) Improving knowledge of antenatal care (ANC) among pregnant women: a field trial in central Java, Indonesia. Asia-Pacific Journal of Public Health 17(1): 3–8. 16044824 10.1177/101053950501700102

[26] Ochako R, Fotso JC, Ikamari L, Khasakhala A (2011) Utilization of maternal health services among young women in Kenya: insights from the Kenya Demographic and Health Survey, 2003. BMC Pregnancy & Childbirth 11: 1. 21214960 10.1186/1471-2393-11-1 PMC3022772

[27] Gwatkin D, Rutstein S, Johnson K, Suliman E, Wagstaff A, et al. (2007) Socio-economic differences in health, nutrition, and population within developing countries: An overview. World Bank. Government of the Netherlands, Swedish International Development Agency. 18293634

[28] Ajwad MI (2007) Attaining the Health and Education Millennium Development Goals in Nepal. Report number 12. Available: http://siteresources.worldbank.org/INTSARREGTOPLABSOCPRO/Resources/AttainingMDGsNepal.pdf Accessed 2014 Mar 09.

[29] Sharma SK, Sawangdee Y, Sirirassamee B (2007) Access to health: women’s status and utilization of maternal health services in Nepal. J Biosoc Sci 39(5): 671–692. 17359562 10.1017/S0021932007001952

[30] UNDP. (2010) The Real Wealth of Nations: Pathways to Human Development. Human Development Report 2010: 20th Anniversary Edition ed. Palgrave Macmillan, Basingstoke, Hampshire, and New York: United Nations Development Programme.

[31] Powell-Jackson T, Neupane BD, Tiwari S, Morrison J, Costello A (2008) Evaluation of the safe delivery incentive programme: Final Report of the Evaluation.

[32] Maine D, Wardlaw TM, Ward VM (1997) Guidelines for monitoring the availability and use of obstetric services. United Nations Children’s Fund.

[33] Abou-Zahr CL, Wardlaw TM (2003) Antenatal Care in Developing Countries: Promises, achievements and missed opportunities. Available: http://www.apcom.org/ids-document/antenatal-caredeveloping-countries-promises-achievements-and-missed-opportunities Accessed 2014 Mar 09.

[34] Kishowar Hossain AH (2010) Utilization of antenatal care services in Bangladesh: an analysis of levels, patterns, and trends from 1993 to 2007. Asia-Pacific Journal of Public Health 22(4): 395–406. 20498123 10.1177/1010539510366177

[35] Witter S, Khadka S, Nath H, Tiwari S (2011) The national free delivery policy in Nepal: early evidence of its effects on health facilities. Health Policy Plan. 26 (suppl 2)ii84–ii91. 10.1093/heapol/czr066 22027923

[36] Karki, Yagya B., Gajanand Agrawal (2008) Effects of Communication Campaigns on the Health Behavior of Women of Reproductive Age in Nepal: Further Analysis of the 2006 Nepal Demographic and Health Survey. Calverton, Maryland, USA: Macro International Inc.

[37] JHPIEGO (2004) Behavior change interventions for Safe Motherhood: common problems unique solutions.

[38] Pant PD (2008) Improvements in maternal health in Nepal: further analysis of the 2006 Nepal Demographic and Health Survey. Population Division, Ministry of Health and Population, Government of Nepal.

[39] Bertrand JT, Escudero G (2002) Compendium of Indicators for Evaluation Reproductive Health Programs Vol 1, No. 6.

